# Adjuvant immunotherapy after neoadjuvant immunochemotherapy and esophagectomy for esophageal squamous cell carcinoma: a real-world study

**DOI:** 10.3389/fimmu.2024.1456193

**Published:** 2024-12-17

**Authors:** Jifeng Feng, Liang Wang, Xun Yang, Qixun Chen

**Affiliations:** ^1^ Department of Thoracic Oncological Surgery, Zhejiang Cancer Hospital, Hangzhou Institute of Medicine (HIM), Chinese Academy of Sciences, Hangzhou, China; ^2^ Key Laboratory of Diagnosis and Treatment Technology on Thoracic Oncology (Lung and Esophagus) of Zhejiang Province, Zhejiang Cancer Hospital, Hangzhou, China

**Keywords:** neoadjuvant immunochemotherapy, adjuvant immunotherapy, esophageal squamous cell carcinoma, disease-free survival, overall survival

## Abstract

**Background:**

The role of immunotherapy in the adjuvant setting seems promising in recent years. As per the findings of the CheckMate 577 trial, patients with esophageal cancer (EC) who had neoadjuvant chemoradiation with residual pathologic disease should be considered adjuvant immunotherapy (AIT). However, it is unknown if individuals with esophageal squamous cell carcinoma (ESCC) who have received neoadjuvant immunochemotherapy (NICT) followed by radical surgery also require AIT.

**Methods:**

A retrospective analysis was performed on the data from patients who underwent NICT and radical surgery for ESCC between 2019 and 2020. To compare disease-free survival (DFS) and overall survival (OS), Kaplan-Meier survival curves were produced. To determine the parameters linked to DFS and OS, a Cox model using hazard ratios (HRs) was completed.

**Results:**

Among the 292 eligible patients, 215 cases with a mean age of 63.3 ± 6.8 years, including 190 (88.4%) men and 25 (11.6%) women, were finally recruited. The percentage of R0 resection was 98.3%. After NICT, 65 (30.2%) patients achieved pathological complete response. AIT was given to 78 (36.3%) patients following radical resection. For all patients, the 3-year DFS and OS were 62.3% and 74.0%, respectively. In terms of 3-year DFS (61.5% vs. 62.8%, P=0.984) or OS (76.9% vs. 72.3%, P=0.384), no statistically significant difference was found between patients with and without AIT. AIT significantly improved survival in patients with ypT+N+ (DFS: 23.9% vs. 38.5%, P=0.036; OS: 37.0% vs. 61.5%, P=0.010), but not in those with ypT0N0 or ypT+N0. It was found that AIT was related to both DFS (HR: 0.297; P<0.001) and OS (HR: 0.321; P=0.001) in patients with ypT+N+.

**Conclusion:**

In ypT+N+ ESCC patients, AIT after NICT followed by radical surgery reduces the recurrence and death, thereby improving the DFS and OS. Randomized controlled trials ought to be conducted to further assess the results of this retrospective investigation.

## Introduction

Ranking 7th and 6th in terms of cancer morbidity and mortality, respectively, esophageal cancer (EC), primarily esophageal adenocarcinoma (EAC) and esophageal squamous cell carcinoma (ESCC), is one of the most prevalent cancer types worldwide (1). The prognosis for EC is still unsatisfactory because of cancer metastasis and recurrence, even with significant efforts in multidisciplinary therapies (2). For individuals with locally advanced disease, neoadjuvant chemoradiotherapy (NCRT) or neoadjuvant chemotherapy (NCT) followed by radical surgery represents the standard treatment (3, 4). Post-surgery, for those with neoadjuvant therapy (NAT), the standard care strategy is observation. Patients confront a high risk of treatment failure, nevertheless, if they do not experience a pathological complete response (PCR) following surgery (5, 6). In order to improve the survival rate, researchers continue to explore more appropriate adjuvant therapies (ATs), such as adjuvant chemotherapy (ACT) or adjuvant chemoradiotherapy (ACRT) (7, 8).

Recently, the prognosis of advanced EC has significantly changed due to immunotherapy, an emerging treatment hotspot (9, 10). Additionally, studies have demonstrated the efficacy and safety of neoadjuvant immunochemotherapy (NICT) for locally advanced EC (11–13). However, further verification is necessary to fully understand the therapeutic response and clinical outcomes of NICT. Furthermore, the need for AT after NICT followed by radical resection has not yet been determined. In particular, a higher risk of recurrence is associated with patients who had pathologic residual disease following NICT and surgery. Therefore, a more appropriate AT should be administered to those patients. The phase III trial CheckMate 577 reported that nivolumab was most beneficial for patients with ESCC, with a disease-free survival (DFS) of 29.7 months. Additionally, following a median follow-up of 2 years, nivolumab was linked to a 31% lower risk of death or recurrence (14).

As NICT is a new treatment mode in recent years, although effective progress has been made in terms of safety and efficacy, there are still uncertainties about AT after surgery due to the lack of data and the fact that relevant studies have not reached the specified prognosis observation time. At present, NICT for EC can achieve good short-term clinical outcomes. There is a lack of evidence to support which AT should be given after NICT. The use of AIT following NICT and surgery is currently not well supported by the literature, raising questions about which patients to treat and how ultimate pathology may affect these clinical decisions. Accordingly, the purpose of this retrospective study was to assess the effectiveness of AIT in patients with ESCC following NICT plus surgery.

## Materials and methods

### Patients

A retrospective analysis was performed on the data from patients who underwent NICT and surgery for ESCC between 2019 and 2020. **Supplementary Figure S1** presents the inclusion criteria. All patients were enrolled in the investigator-initiated clinical trials (IITs). The following were the exclusion criteria: (1) pathological diagnosis of non-ESCC; (2) received non-radical resection; (3) in combination with NCRT; (4) surgical-related mortality; (5) accompanied or previously accompanied by cancers at other sites; (6) received ACRT after radical resection; and (7) incomplete clinical data or follow-up. Finally, 215 patients were included in the analysis. The 8th AJCC/UICC TNM classification system was used in this study (15). The Ethics Committee of Zhejiang Cancer Hospital gave its approval (IRB-2020-320) and the research was carried out in compliance with the Helsinki Declaration.

### Treatment and follow-up

Two NICT cycles were administered to eligible patients in this investigation, with 200 mg of camrelizumab, tislelizumab, or sintilimab, 2mg/Kg of pembrolizumab, or 3mg/Kg of nivolumab, administered on day 1, albumin-paclitaxel (120 mg/m^2^) administered on days 1 and 8, and carboplatin [5 mg/ml/min on the basis of the area under the curve (AUC)] administered on day 1 of each 21-day cycle. McKeown or Ivor Lewis, as a classic surgical procedure, was typically carried out 4-6 weeks after the end of the last NICT cycle (16). In principle, two-field lymph node (LN) dissection is indicated when tumors are located at the middle to lower thoracic esophagus, while three-field LN dissection is applied for upper thoracic tumors. There is currently no agreement on AT in situations where radical surgery is needed after NICT. The CheckMate 577 trial suggests that AIT may be beneficial for patients following NCRT (14). Accordingly, AIT was advised for patients who did not obtain PCR, according to the EC expert agreement on perioperative immunotherapy (17). The duration time for patients to choose postoperative AIT was 1-2 years, but it is not mandatory, mainly based on the postoperative pathological results. The latest follow-up period was completed in December 2023.

### Statistical analysis

The chi-square or Fisher’s exact tests were used to compare categorical variables. The Student t-test was utilized for normally distributed continuous variables, whereas the Mann-Whitney U-test was employed for those variables with a non-normal distribution. Patient survival was compared according to AIT using the Kaplan-Meier method. To determine the parameters linked to DFS and overall survival (OS), a Cox proportional hazard model using hazard ratios (HRs) was completed. SPSS 20.0 was used to perform all two-sided statistical tests, with statistical significance indicated by P values <0.05.

## Results

### Patients characteristics

**Table 1** summarizes patient characteristics. After all, 215 cases with a mean age of 63.3 ± 6.8 years, 190 men (88.4%), and 25 women (11.6%) were selected from the 292 eligible patients. Regarding NICT, there were 12 (5.6%), 27 (12.6%), 118 (54.8%), 43 (20.0%), and 15 (7.0%) patients who were treated with nivolumab, pembrolizumab, camrelizumab, tislelizumab, and sintilimab, respectively. The R0 resection rate was 98.3%. After NICT, 65 (30.2%) cases achieved PCR. A total of 78 (36.3%) cases received AIT, including 5 (6.4%) of nivolumab, 14 (18.0%) of pembrolizumab, 33 (42.3%) of camrelizumab, 21 (26.9%) of tislelizumab, and 5 (6.4%) of sintilimab, respectively. After NICT, 83 (83/137, 60.6%) patients in the non-AIT group and 67 (67/78, 85.9%) cases in the AIT group had any residual disease. Among all the patients, 39 (39/78, 50.0%) cases in the AIT group and 46 (46/137, 33.6%) cases in the non-AIT cohort had any residual nodal disease. Patients who did not get AIT had a greater rate of PCR (P<0.001), while those who got AIT had higher ypT (P<0.001), ypN (P=0.023), and ypTNM (P<0.001) stages.

**Table 1:** Characteristics in all patients with ESCC receiving NICT.

	Total (n=215)	Non-AIT (n=137)	AIT (n=78)	P-value
Sex (n, %)				0.360
female	25 (11.6)	18 (13.1)	7 (9.0)	
male	190 (88.4)	119 (86.9)	71 (91.0)	
Age (median, Q1-3, years)	64 (57-69)	64 (57-68)	64.5 (58-69)	0.646
BMI (median, Q1-3, Kg/m^2^)	21.7 (20.2-22.6)	21.5 (20.4-22.5)	21.7 (20.1-23.1)	0.632
Tumor location (n, %)				0.412
upper	20 (9.3)	15 (10.9)	5 (6.4)	
middle	124 (57.7)	80 (58.4)	44 (56.4)	
lower	71 (33.0)	42 (30.7)	29 (37.2)	
Differentiation (n, %)				0.002
well	49 (22.8)	39 (28.5)	10 (12.8)	
moderate	95 (44.2)	63 (46.0)	32 (41.0)	
poor	71 (33.0)	35 (25.5)	36 (46.2)	
Vessel invasion (n, %)	45 (20.9)	20 (14.6)	25 (32.1)	0.002
Perineural invasion (n, %)	47 (21.9)	24 (17.5)	23 (29.5)	0.041
Tumor length (median, Q1-3, cm)	1.90 (0.0-3.0)	1.20 (0.0-2.6)	2.75 (1.2-4.0)	<0.001
Immunotherapy (n, %)				0.052
nivolumab	12 (5.6)	7 (5.1)	5 (6.4)	
pembrolizumab	27 (12.6)	13 (9.5)	14 (17.9)	
camrelizumab	118 (54.8)	85 (62.0)	33 (42.3)	
tislelizumab	43 (20.0)	22 (16.1)	21 (26.9)	
sintilimab	15 (7.0)	10 (7.3)	5 (6.4)	
Surgical method (n, %)				0.386
McKeown	185 (86.0)	120 (87.6)	65 (83.3)	
Ivor-Lewis	30 (14.0)	17 (12.4)	13 (16.7)	
PCR (n, %)	65 (30.2)	54 (39.4)	11 (14.1)	<0.001
ypT stage (n, %)				<0.001
T0	65 (30.2)	54 (39.4)	11 (14.1)	
T1	35 (16.3)	24 (17.5)	11 (14.1)	
T2	38 (17.7)	27 (19.7)	11 (14.1)	
T3	57 (26.5)	25 (18.2)	32 (41.0)	
T4	20 (9.3)	7 (5.2)	13 (16.7)	
ypN stage (n, %)				0.023
N0	130 (60.5)	91 (66.4)	39 (50.0)	
N1	48 (22.3)	27 (19.7)	21 (26.9)	
N2	26 (12.1)	16 (11.7)	10 (12.8)	
N3	11 (5.1)	3 (2.2)	8 (10.3)	
ypTNM stage (n, %)				<0.001
stage 0	65 (30.2)	54 (39.4)	11 (14.1)	
stage I	42 (19.5)	29 (21.2)	13 (16.7)	
stage II	20 (9.3)	7 (5.1)	13 (16.7)	
stage III	64 (29.8)	39 (28.5)	25 (32.1)	
stage IV	24 (11.2)	8 (5.8)	16 (20.5)	
Total LNs (median, Q1-3, n)	20 (16-26)	19 (15-25)	22 (18-29)	0.009
Positive LNs (median, Q1-3, n)	0 (0-1)	0 (0-1)	1 (0-2)	0.001
Negative LNs (median, Q1-3, n)	19 (15-26)	18 (14-25)	20 (16-26)	0.130

ESCC, esophageal squamous cell carcinoma; NICT, neoadjuvant immunochemotherapy; AIT, adjuvant immunotherapy; BMI, body mass index; SD, standard deviation; TNM, tumor node metastasis; PCR, pathological complete response; LN, lymph node.

### Survival analyses for DFS and OS

**Figure 1:**
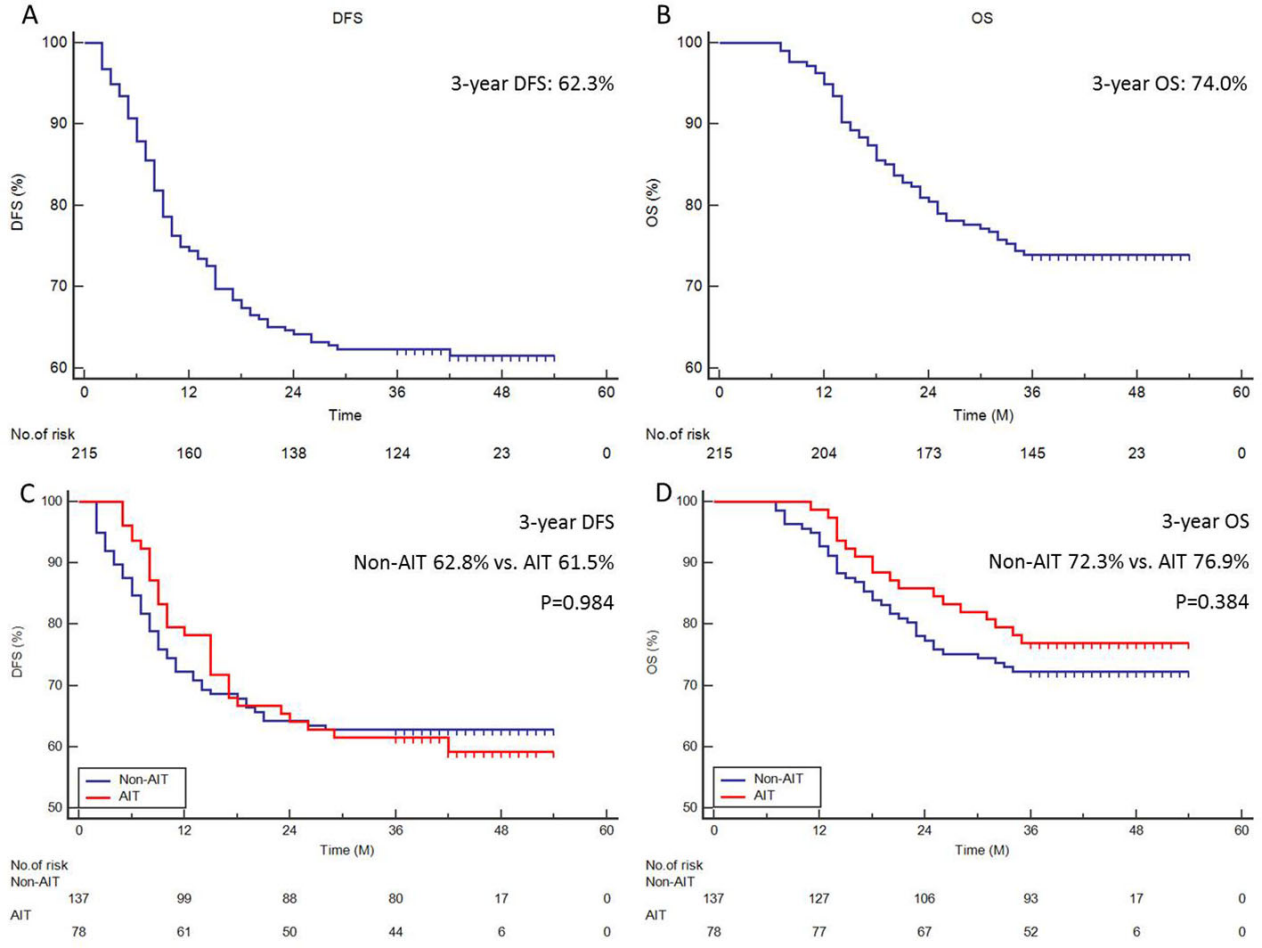
Survival analyses. The 3-year DFS **(A)** or 3-year OS **(B)** of all cohorts. The 3-year DFS **(C)** or 3-year OS **(D)** in patients with and without AIT.

In total, 82 (38.1%) cases had recurrence, and 56 (26.0%) cases died. Patients were classified as having a local recurrence or a distant recurrence based on their original presentation. Following treatment, 49 patients (59.8%) experienced distant recurrence, which included non-regional LN metastasis; in contrast, 33 patients (40.2%) experienced local recurrence, which included locoregional LN metastasis and anastomotic site recurrence. However, upon further analysis, the result revealed that AIT can effectively reduce distant recurrence (14.1% vs. 27.7%, P=0.022), but not for local recurrence (19.2% vs. 13.1%, P=0.233) (**Supplementary Figure S2**). The median follow-up period was 40 months. The 3-year DFS and OS were 62.3% (**Figure 1A**) and 74.0% (**Figure 1B**) in all patients, respectively. There was no statistically significant difference in the 3-year DFS (61.5% vs. 62.8%, P=0.984, **Figure 1C**) or 3-year OS (76.9% vs. 72.3%, P=0.384, **Figure 1D**) between patients with and without AIT.

### Subgroup analysis in survival

**Figure 2:**
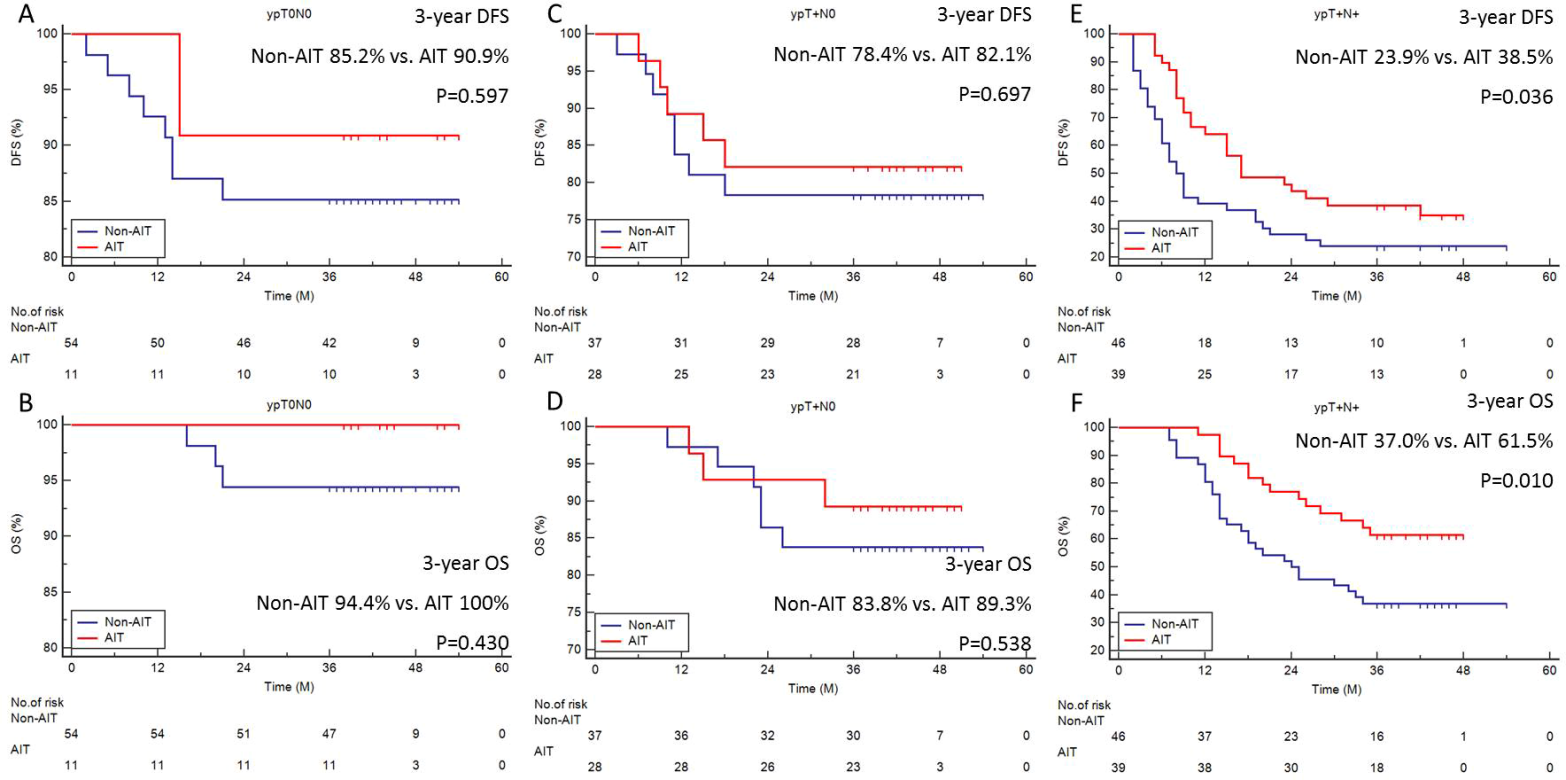
Subgroup analyses of survival. The 3-year DFS **(A)** or OS **(B)** in ypT0N0. The 3-year DFS **(C)** or OS **(D)** in ypT+N0. The 3-year DFS **(E)** or OS **(F)** in ypT+N+.

Subgroup analysis of survival (DFS and OS) was carried out based on ypT0N0, ypT+N0, and ypT+N+ since AIT was typically carried out according to the pathologic status after surgery. For individuals with ypT0N0 (**Figures 2A, B**) and ypT+N0 (**Figures 2C, D**), the survival benefit of AIT was not statistically significant, but it was significant in those with ypT+N+ (3-year DFS: 23.9% vs. 38.5%, P=0.036, **Figure 2E**; 3-year OS: 37.0% vs. 61.5%, P=0.010, **Figure 2F**).

### Patient characteristics in ypT+N+

**Table 2** provides a summary of the features of ypT+N+ patients. Tumor length (P=0.012), positive LNs (P=0.007), and ypTNM stage (P=0.044) were different between the two groups, whereas other clinical characteristics were not significantly different between the two groups. Even though patients with AIT had longer tumor lengths, more metastatic LNs, and higher ypTNM stages, the results showed that these cases had a better prognosis than those without AIT, which further demonstrated the positive effect of AIT.

**Table 2:** Characteristics in ypT+N+ patients with ESCC receiving NICT.

	Total (n=85)	Non-AIT (n=46)	AIT (n=39)	P-value
Sex (n, %)				0.347
female	12 (14.1)	8 (17.4)	4 (10.3)	
male	73 (85.9)	38 (82.6)	35 (89.7)	
Age (median, Q1-3, years)	64 (57-69)	63 (57-68)	64 (57-69)	0.744
BMI (median, Q1-3, Kg/m^2^)	21.8 (19.8-23.1)	21.6 (19.6-22.6)	21.8 (20.2-23.6)	0.359
Tumor location (n, %)				0.419
upper	10 (11.8)	5 (10.9)	5 (12.8)	
middle	37 (43.5)	23 (50.0)	14 (35.9)	
lower	38 (44.7)	18 (39.1)	20 (51.3)	
Differentiation (n, %)				0.204
well	15 (17.6)	9 (19.6)	6 (15.4)	
moderate	33 (38.8)	21 (45.7)	12 (30.8)	
poor	37 (43.6)	16 (34.7)	21 (53.8)	
Vessel invasion (n, %)	27 (20.9)	12 (26.1)	15 (38.5)	0.222
Perineural invasion (n, %)	27 (31.8)	14 (30.4)	13 (33.3)	0.775
Tumor length (median, Q1-3, cm)	3.00 (2.00-4.20)	2.80 (1.88-3.78)	3.20 (2.50-4.60)	0.012
Immunotherapy (n, %)				0.097
nivolumab	4 (4.7)	1 (2.2)	3 (7.7)	
pembrolizumab	14 (16.5)	6 (13.0)	8 (20.5)	
camrelizumab	44 (51.8)	30 (65.2)	14 (35.9)	
tislelizumab	16 (18.8)	6 (13.0)	10 (25.6)	
sintilimab	7 (8.2)	3 (6.6)	4 (10.3)	
Surgical method (n, %)				0.082
McKeown	68 (80.0)	40 (87.0)	28 (71.8)	
Ivor-Lewis	17 (20.0)	6 (13.0)	11 (28.2)	
ypT stage (n, %)				0.055
T1	8 (9.4)	7 (15.2)	1 (2.6)	
T2	23 (27.1)	15 (32.6)	8 (20.5)	
T3	37 (43.5)	18 (39.1)	19 (48.7)	
T4	17 (20.0)	6 (13.0)	11 (28.2)	
ypN stage (n, %)				0.145
N1	48 (56.5)	27 (58.7)	21 (53.8)	
N2	26 (30.6)	16 (34.8)	10 (25.7)	
N3	11 (12.9)	3 (6.5)	8 (20.5)	
ypTNM stage (n, %)				0.044
stage IIIA	19 (22.4)	13 (28.3)	6 (15.4)	
stage IIIB	42 (49.4)	15 (54.3)	17 (43.6)	
stage IVA	24 (28.2)	8 (17.4)	16 (41.0)	
Total LNs (median, Q1-3, n)	21 (17-29)	20 (15-27)	22 (20-30)	0.064
Positive LNs (median, Q1-3, n)	2 (1-4)	1 (0-3)	2 (1-6)	0.007
Negative LNs (median, Q1-3, n)	19 (15-26)	18 (14-26)	20 (15-26)	0.466

ESCC, esophageal squamous cell carcinoma; NICT, neoadjuvant immunochemotherapy; AIT, adjuvant immunotherapy; BMI, body mass index; SD: standard deviation; TNM, tumor node metastasis; PCR, pathological complete response; LN, lymph node.

### Prognostic factors for survival in ypT+N+

**Supplementary Figures S3A, B** presents the findings from multivariable analysis in ypT+N+ individuals. In patients with ypT+N+ ESCC, AIT was linked to survival following NICT plus radical surgery (DFS: HR=0.297, P<0.001; OS: HR=0.321, P=0.001). Consequently, AIT following NICT and surgery lowers the risk of death and recurrence in ypT+N+ ESCC patients, improving their prognosis. The Sankey diagrams regarding relations among AIT, ypTNM stages, and prognosis for all patients and ypT+N+ are shown in **Supplementary Figures S3C, D**.

### Correlations between preoperative efficacy and prognosis

Further analysis was done on the correlation between prognosis following NICT and clinical stage decline. Any T and/or N decline in patients after NICT was regarded as a clinical stage decline in this study. A clinical stage decline was observed in 170 (79.1%) patients, while 57 (67.1%) of the ypT+N+ patients also showed a clinical stage decline. Clinical stage decline was strongly correlated with DFS and OS in both the total and ypT+N+ patients. The survival benefit was significant in those with clinical stage decline (Total: 3-year DFS: 66.5% vs. 46.7%, P=0.009, **Supplementary Figure S4A**; 3-year OS: 78.2% vs. 57.8%, P=0.008, **Supplementary Figure S4B**; ypT+N+: 3-year DFS: 38.6% vs. 14.3%, P=0.013, **Supplementary Figure S4C**; 3-year OS: 56.1% vs. 32.1%, P=0.016, **Supplementary Figure S4D**).

## Discussion

For locally advanced EC, NAT is now the accepted therapeutic option in certain countries. For patients with localized EC, the National Comprehensive Cancer Network (NCCN) guidelines suggest NCRT (3). On the other hand, NCT is advised by Japanese recommendations for those with resectable stage II or III thoracic EC (4). Due to immunotherapy as an emerging treatment modality, there is currently a lack of information to enlighten clinicians of its potential benefits to the point of care and help guide clinical decision making. In our investigation, 30.2% of patients who had PCR after NICT demonstrated a respectable survival rate (3-year DFS: 86.2% and 3-year OS: 95.4%). Therefore, AIT did not significantly improve survival in patients with PCR (ypT0N0), and follow-up without further treatment was feasible. Regrettably, patients with a residual pathologic viable lesion have a poor prognosis, and PCR is frequently not obtained in most cases (5, 6). It needs more research to fully confirm how beneficial AIT is for individuals who have had surgery and NICT. In terms of 3-year DFS or 3-year OS, there was no statistically significant difference between those with and without AIT. Nonetheless, individuals with ypT+N+ showed a significant survival benefit from AIT (DFS: P=0.036; OS: P=0.010). It was also found that AIT was related to both DFS (HR: 0.297; P<0.001) and OS (HR: 0.321; P=0.001) in patients with ypT+N+.

It is generally acknowledged that NAT is useful for EC; nevertheless, the function of AT is still not really clear. AIT has been shown to enhance DFS in patients who had NCRT and surgical resection for EC, according to the recent Checkmate 577 trial (14). However, its use for those with NICT is limited. The administration of postoperative ACT to those with stage II or III ESCC who had NAT with surgery is not well-supported by available data, according to the Japan Esophageal Society’s practice guidelines (18). Furthermore, the practice guidelines on multimodality treatment for EC published by the Society for Thoracic Surgeons also advise against providing the optimal treatment to node-positive patients who have already had multimodality therapy (19). In addition, a multiinstitutional study discovered that the rate of AT ranged from 3.2% to 50% in real clinical practice, indicating that AT is administered on a basis to numerous patients and varies greatly throughout clinicians and institutions (20).

The effects of AT following NAT and surgery have been investigated in a number of retrospective researches. Kim et al. (21) revealed that ACT after NCRT has been shown to be viable; however, the study’s conclusions might have been impacted by the limited sample size. Mokda et al. (22) came to the same conclusion, with a small number of postoperative ACTs despite having up to 10,000 patients who underwent NCRT prior to surgery. According to studies published by Glatz et al. (23) and Kamarajah et al. (24), OS can be enhanced by ACT administered after NCT. Nonetheless, other research also presents differing findings. According to a multicenter cohort trial, patients with R1 resection were the only ones who benefited from ACT administered after NCT for EA, with no improvement in prognosis (25). Similar findings were also made by studies conducted by Bott et al. (26) and Li et al. (27). According to a recent meta-analysis, AT following NAT with negative resection margins improves 1- and 5-year OS with moderate to high confidence of evidence; however, because these outcomes are not widely reported, the benefit for DFS is still unknown (8). Patients contemplating AT should be advised on benefits versus morbidity because the benefit of AT is frequently minimal (7).

The primary NAT employed in these investigations was NCRT, while the primary pathogenic form of EC examined was EA. The advantages of our study are demonstrated by the fact that, in contrast to earlier research, it examined a uniform pathology of ESCC and offered comprehensive information on the NICT. A further advantage of this research was that the follow-up period of three years and a specific sample size were chosen to provide a good predictive value for the prognosis analysis. According to earlier research, individuals with pathologic node-positive (ypN+) conditions greatly benefit from AT. Samson et al. (28) found that ACT led to a better median OS in patients with ypN+ from the National Cancer Database who had NAT plus surgery. Semenkovich et al. (20) indicated that patients with ypN+ who underwent NAT and surgery in a multicenter retrospective analysis who got ACT had a longer median OS compared to those who did not examine patients. In patients with ypT0N0 or ypT+N0, Burt and colleagues found that ACT did not significantly lower the probability of mortality. On the other hand, among individuals with ypTanyN+, ACT was linked to a 30% decrease in the risk of death in the whole cohort (29). According to Park et al.’s research, ACT following NAT and surgery improves the OS in those with ypT+N+ ESCC by reducing distant metastases (30). In our research, patients with ypT+N+ clearly benefit from AIT, but those with ypT+N0 or ypT0N+ did not demonstrate any benefit to survival. Compared to those with ypT+N0 or ypT0N+, the impact of additionally AIT may theoretically be more pronounced in those with ypT+N+ since they have a lower survival rate.

Although the primary finding of this study indicates that AIT improves DFS and OS in ypT+N+ patients following NICT and surgery, we believe that AIT cannot be consistently given to all ypT+N+ ESCC patients uniformly. Carefully assessing the patient’s status following NICT and esophagectomy is necessary, as is weighing the advantages of AIT in terms of survival against the danger of recurrence. Furthermore, clinicians must find suitable patients with tolerance status so they can receive AIT, and further research is needed to determine the standards for candidate screening. In addition, efforts must be undertaken to lower postoperative complications and increase long-term survival because early postoperative morbidity and death are barriers to AIT.

There is currently no agreement on AIT in situations where R0 resection is required following NICT. Patients who achieved a PCR in the CheckMate 577 trial with the 5-year OS of 47-72% were excluded because they were thought to be at low risk of recurrence (14). Nonetheless, between 17% and 39% of these individuals later developed recurrences, with locoregional recurrence being the primary treatment failure pattern. In the current study, therefore, the purpose was to assess the effectiveness of AIT in patients with ESCC following NICT plus surgery as well as in those with PCR. The study indicated that AIT significantly improved both DFS and OS in patients with ypT+N+, but not in those with PCR.

Although there are no long-term follow-ups from trials, clinical evidence indicates that NICT with R0 resection may be an appealing therapeutic option for individuals with ESCC. Based on Checkmate 577 results, some national guidelines have changed their recommendations for adjuvant nivolumab for non-PCR patients after surgery following NCRT (14). Compared to historical data, Mamdani et al. (NCT02639065) demonstrated that adjuvant durvalumab significantly improved the 1-year recurrence-free survival for those with locally progressed EC and pathologically remaining disease after R0 resection following NCRT (31). However, the findings reported by Park et al. (NCT02520453) differ from the Checkmate 577 trial and Mamdani’s. The results revealed that there was no significant difference in DFS or OS between the two groups (32). Furthermore, there are no guidelines to recommend how many courses of AIT are required. In another research (NCT04437212), Toripalimab was administered every three weeks for four cycles as adjuvant treatment (33). However, the authors stated that based on existing evidence and standards, four cycles of AIT may not be sufficient for those without PCR after NAT. Therefore, further research is needed to explore the optimal AT for patients who have undergone NICT and surgery.

This study has some limitations. Firstly, this is a retrospective cohort study from a single institution. However, the current study is of great significance in the absence of sufficient evidence-based medical evidence. A retrospective analysis was used to examine past cases in order to obtain evidence, as there haven’t been any from these clinical studies to date. Secondly, there are a variety of immune drugs, and there may be differences in prognosis between different immune drugs. However, our findings showed that there was no statistical difference in the characteristics and prognosis of different immune agents. Finally, the decision for patients to receive AIT, with some selectivity, was largely determined by clinicians. However, in patients with ypT+N+, there was no significant difference between patients with or without AIT. Consequently, more randomized controlled clinical trials are necessary to determine the indications and treatment plan for AIT.

In summary, after NICT and surgery for ESCC, AIT increased DFS and OS in ypT+N+ patients. Since these individuals are able to tolerate the additional treatment, AIT may be a viable alternative for them. However, additional trials should be conducted to better examine the results of this retrospective investigation.

## Data Availability

The original contributions presented in the study are included in the article/**Supplementary Material**. Further inquiries can be directed to the corresponding author.

## Supplementary material

Supplementary Figure 1 The inclusion criteria of current study.

Supplementary Figure 2 Correlation between AIT and recurrence. AIT can effectively reduce distant recurrence (14.1% vs. 27.7%, P=0.022), but not for local recurrence (19.2% vs. 13.1%, P=0.233).

Supplementary Figure 3 Multivariable analysis in ypT+N+ individuals. Parameters linked to DFS **(A)** or OS **(B)** in patients with ypT+N+ ESCC. The Sankey diagrams regarding relations among AIT, ypTNM stages, and prognosis for all cohorts **(C)** or those with ypT+N+ **(D)**.

Supplementary Figure 4 Survival grouped by clinical stage decline. The 3-year DFS **(A)** or 3-year OS **(B)** of all cohorts with and without clinical stage decline. The 3-year DFS **(C)** or 3-year OS **(D)** in ypT+N+ patients with and without clinical stage decline.

## References

[1] Sung H Ferlay J Siegel RL Laversanne M Soerjomataram I Jemal A. Global cancer statistics 2020: GLOBOCAN estimates of incidence and mortality worldwide for 36 cancers in 185 countries. CA Cancer J Clin. (2021) 71:209–49. doi: 10.3322/caac.21660 33538338

[2] Zhang Y Gao J Zheng A Yang H Li J Wu S. Definition and risk factors of early recurrence based on affecting prognosis of esophageal squamous cell carcinoma patients after radical resection. Transl Oncol. (2021) 14:101066. doi: 10.1016/j.tranon.2021.101066 33744728 PMC7985560

[3] Shapiro J van Lanschot JJB Hulshof MCCM van Berge Henegouwen MI Wijnhoven BPL. Neoadjuvant chemoradiotherapy plus surgery versus surgery alone for oesophageal or junctional cancer (CROSS): long-term results of a randomised controlled trial. Lancet Oncol. (2015) 16:1090–8. doi: 10.1016/S1470-2045(15)00040-6 26254683

[4] Ando N Kato H Igaki H Shinoda M Ozawa S Shimizu H. A randomized trial comparing postoperative adjuvant chemotherapy with cisplatin and 5-fluorouracil versus preoperative chemotherapy for localized advanced squamous cell carcinoma of the thoracic esophagus (JCOG9907). Ann Surg Oncol. (2012) 19:68–74. doi: 10.1245/s10434-011-2049-9 21879261

[5] Hipp J Kuvendjiska J Hillebrecht HC Herrmann S Timme-Bronsert S Fichtner-Feigl S. Oncological recurrence following pathological complete response after neoadjuvant treatment in patients with esophageal cancer - a retrospective cohort study. Langenbecks Arch Surg. (2023) 408:363. doi: 10.1007/s00423-023-03100-2 37721586 PMC10506930

[6] Blum Murphy M Xiao L Patel VR Maru DM Correa AM G Amlashi F. Pathological complete response in patients with esophageal cancer after the trimodality approach: The association with baseline variables and survival-The University of Texas MD Anderson Cancer Center experience. Cancer. (2017) 123:4106–13. doi: 10.1002/cncr.v123.21 28885712

[7] Rucker AJ Raman V Jawitz OK Voigt SL Harpole DH D'Amico TA. The impact of adjuvant therapy on survival after esophagectomy for node-negative esophageal adenocarcinoma. Ann Surg. (2022) 275:348–55. doi: 10.1097/SLA.0000000000003886 PMC7502525 32209899

[8] Lee Y Samarasinghe Y Lee MH Thiru L Shargall Y Finley C. Role of adjuvant therapy in esophageal cancer patients after neoadjuvant therapy and esophagectomy: A systematic review and meta-analysis. Ann Surg. (2022) 275:91–8. doi: 10.1097/SLA.0000000000005227 34596073

[9] Kojima T Shah MA Muro K Francois E Adenis A Hsu CH. Randomized phase III KEYNOTE-181 study of pembrolizumab versus chemotherapy in advanced esophageal cancer. J Clin Oncol. (2020) 38:4138–48. doi: 10.1200/JCO.20.01888 33026938

[10] Kato K Cho BC Takahashi M Okada M Lin CY Chin K. Nivolumab versus chemotherapy in patients with advanced oesophageal squamous cell carcinoma refractory or intolerant to previous chemotherapy (ATTRACTION-3): a multicentre, randomised, open-label, phase 3 trial. Lancet Oncol. (2019) 20:1506–17. doi: 10.1016/S1470-2045(19)30626-6 31582355

[11] Yang W Xing X Yeung SJ Wang S Chen W Bao Y. Neoadjuvant programmed cell death 1 blockade combined with chemotherapy for resectable esophageal squamous cell carcinoma. J Immunother Cancer. (2022) 10:e003497. doi: 10.1136/jitc-2021-003497 35022193 PMC8756283

[12] Ge F Huo Z Cai X Hu Q Chen W Lin G. Evaluation of clinical and safety outcomes of neoadjuvant immunotherapy combined with chemotherapy for patients with resectable esophageal cancer: A systematic review and meta-analysis. JAMA Netw Open. (2022) 5:e2239778. doi: 10.1001/jamanetworkopen.2022.39778 36322089 PMC9631099

[13] Wang Z Shao C Wang Y Duan H Pan M Zhao J. Efficacy and safety of neoadjuvant immunotherapy in surgically resectable esophageal cancer: A systematic review and meta-analysis. Int J Surg. (2022) 104:106767. doi: 10.1016/j.ijsu.2022.106767 35840049

[14] Kelly RJ Ajani JA Kuzdzal J Zander T Van Cutsem E Piessen G. Adjuvant nivolumab in resected esophageal or gastroesophageal junction cancer. N Engl J Med. (2021) 384:1191–203. doi: 10.1056/NEJMoa2032125 33789008

[15] Rice TW Ishwaran H Hofstetter WL Hofstetter WL Apperson-Hansen C Blackstone EH. Recommendations for pathologic staging (pTNM) of cancer of the esophagus and esophagogastric junction for the 8th edition AJCC/UICC staging manuals. Dis Esophagus. (2016) 29:897–905. doi: 10.1111/dote.2016.29.issue-8 27905172 PMC5591444

[16] Jezerskyte E Saadeh LM Hagens ERC Sprangers MAG Noteboom L van Laarhoven HWM. Long-term health-related quality of life after McKeown and Ivor Lewis esophagectomy for esophageal carcinoma. Dis Esophagus. (2020) 33:doaa022. doi: 10.1093/dote/doaa022 32444879 PMC7672202

[17] Kang X Qin J Zhang R Wang Z Zheng Q Li Y. 2021 NCC/CATS/CSTCVS/STM expert consensus on perioperative immunotherapy for esophageal cancer. Ann Esophagus. (2021) 4:33. doi: 10.21037/aoe-21-64

[18] Kitagawa Y Uno T Oyama T Kato K Kato H Kawakubo H. Esophageal cancer practice guidelines 2017 edited by the Japan esophageal society: part 2. Esophagus. (2019) 16:25–43. doi: 10.1007/s10388-018-0642-8 30171414 PMC6510875

[19] Little AG Lerut AE Harpole DH Hofstetter WL Mitchell JD Altorki NK. The Society of Thoracic Surgeons practice guidelines on the role of multimodality treatment for cancer of the esophagus and gastroesophageal junction. Ann Thorac Surg. (2014) 98:1880–5. doi: 10.1016/j.athoracsur.2014.07.069 25262396

[20] Semenkovich TR Subramanian M Yan Y Hofstetter WL Correa AM Cassivi SD. Adjuvant therapy for node-positive esophageal cancer after induction and surgery: A multisite study. Ann Thorac Surg. (2019) 108:828–36. doi: 10.1016/j.athoracsur.2019.04.099 PMC6904117 31229485

[21] Kim GJ Koshy M Hanlon AL Horiba MN Edelman MJ Burrows WM. The benefit of chemotherapy in esophageal cancer patients with residual disease after trimodality therapy. Am J Clin Oncol. (2016) 39:136–41. doi: 10.1097/COC.0000000000000036 PMC5613244 24487417

[22] Mokdad AA Yopp AC Polanco PM Mansour JC Reznik SI Heitjan DF. Adjuvant chemotherapy vs postoperative observation following preoperative chemoradiotherapy and resection in gastroesophageal cancer: A propensity score-matched analysis. JAMA Oncol. (2018) 4:31–8. doi: 10.1001/jamaoncol.2017.2805 PMC5833647 28975352

[23] Glatz T Bronsert P Schäfer M Kulemann B Marjanovic G Sick O. Perioperative platin-based chemotherapy for locally advanced esophagogastric adenocarcinoma: Postoperative chemotherapy has a substantial impact on outcome. Eur J Surg Oncol. (2015) 41:1300–7. doi: 10.1016/j.ejso.2015.07.010 26253194

[24] Kamarajah SK Markar SR Phillips AW Kunene V Fackrell D Salti GI. Survival benefit of adjuvant chemotherapy following neoadjuvant therapy and oesophagectomy in oesophageal adenocarcinoma. Eur J Surg Oncol. (2022) 48:1980–7. doi: 10.1016/j.ejso.2022.05.014 35718676

[25] Papaxoinis G Kamposioras K Weaver JMJ Kordatou Z Stamatopoulou S Germetaki T. The role of continuing perioperative chemotherapy postsurgery in patients with esophageal or gastroesophageal junction adenocarcinoma: a multicenter cohort study. J Gastrointest Surg. (2019) 23:1729–41. doi: 10.1007/s11605-018-04087-8 30671799

[26] Bott RK Beckmann K Zylstra J Wilkinson MJ Knight WRC Baker CR. Adjuvant therapy following neoadjuvant chemotherapy and surgery for oesophageal adenocarcinoma in patients with clear resection margins. Acta Oncol. (2021) 60:672–80. doi: 10.1080/0284186X.2021.1885057 33586602

[27] Li K Hao W Liu X Li Y Sun H Liu S. The role of adjuvant chemotherapy in the treatment of esophageal squamous cell carcinoma after neoadjuvant chemotherapy. J Cancer. (2023) 14:3130–8. doi: 10.7150/jca.84484 PMC10583580 37859815

[28] Samson P Puri V Lockhart AC Robinson C Broderick S Patterson GA. Adjuvant chemotherapy for patients with pathologic node-positive esophageal cancer after induction chemotherapy is associated with improved survival. J Thorac Cardiovasc Surg. (2018) 156:1725–35. doi: 10.1016/j.jtcvs.2018.05.100 30054137

[29] Burt BM Groth SS Sada YH Farjah F Cornwell L Sugarbaker DJ. Utility of adjuvant chemotherapy after neoadjuvant chemoradiation and esophagectomy for esophageal cancer. Ann Surg. (2017) 266:297–304. doi: 10.1097/SLA.0000000000001954 27501170

[30] Park SY Kim HK Jeon YJ Lee J Cho JH Choi YS. The role of adjuvant chemotherapy after neoadjuvant chemoradiotherapy followed by surgery in patients with esophageal squamous cell carcinoma. Cancer Res Treat. (2023) 55:1231–9. doi: 10.4143/crt.2022.1417 PMC10582531 37114475

[31] Mamdani H Schneider B Perkins SM Burney HN Kasi PM Abushahin LI. A phase II trial of adjuvant durvalumab following trimodality therapy for locally advanced esophageal and gastroesophageal junction adenocarcinoma: a big ten cancer research consortium study. Front Oncol. (2021) 17:736620. doi: 10.3389/fonc.2021.736620 PMC8484871 34604072

[32] Park S Sun JM Choi YL Oh D Kim HK Lee T. Adjuvant durvalumab for esophageal squamous cell carcinoma after neoadjuvant chemoradiotherapy: a placebo-controlled, randomized, double-blind, phase II study. ESMO Open. (2022) 7:100385. doi: 10.1016/j.esmoop.2022.100385 35158205 PMC8850741

[33] Xu X Sun Z Liu Q Zhang Y Shen L Zhang C. Neoadjuvant chemoradiotherapy combined with sequential perioperative toripalimab in locally advanced esophageal squamous cell cancer. J Immunother Cancer. (2024) 12:e008631. doi: 10.1136/jitc-2023-008631 38458635 PMC10921522

