# Supplementary figures and images for: Adjuvant immunotherapy after neoadjuvant immunochemotherapy and esophagectomy for esophageal squamous cell carcinoma: a real-world study
Source: Front Immunol. 2024 Dec 17;15:1456193. doi: 10.3389/fimmu.2024.1456193 (PMC11685212; doi:10.3389/fimmu.2024.1456193)

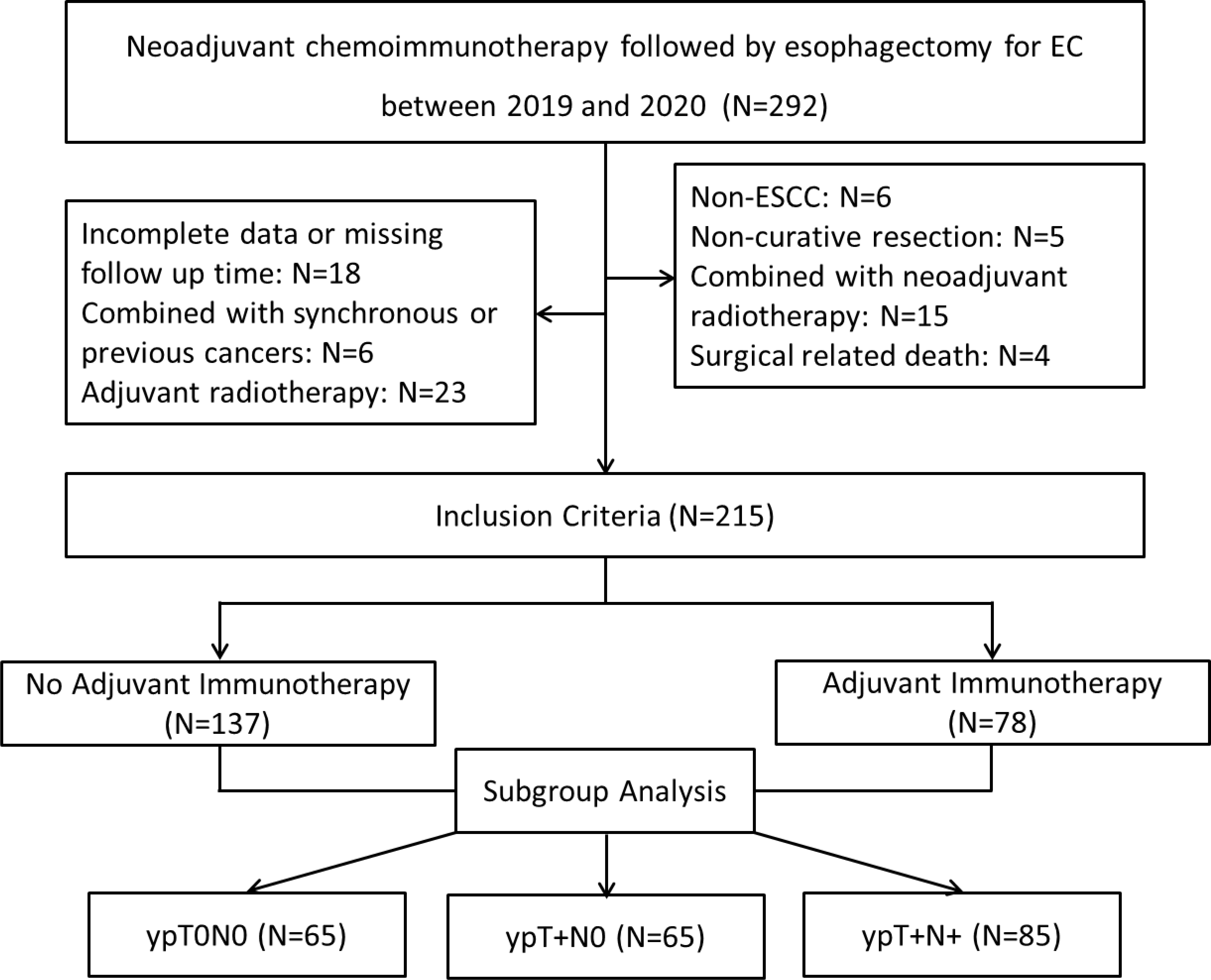

Supplement: Supplementary Figure 1 — The inclusion criteria of current study. [file Image1.jpeg]

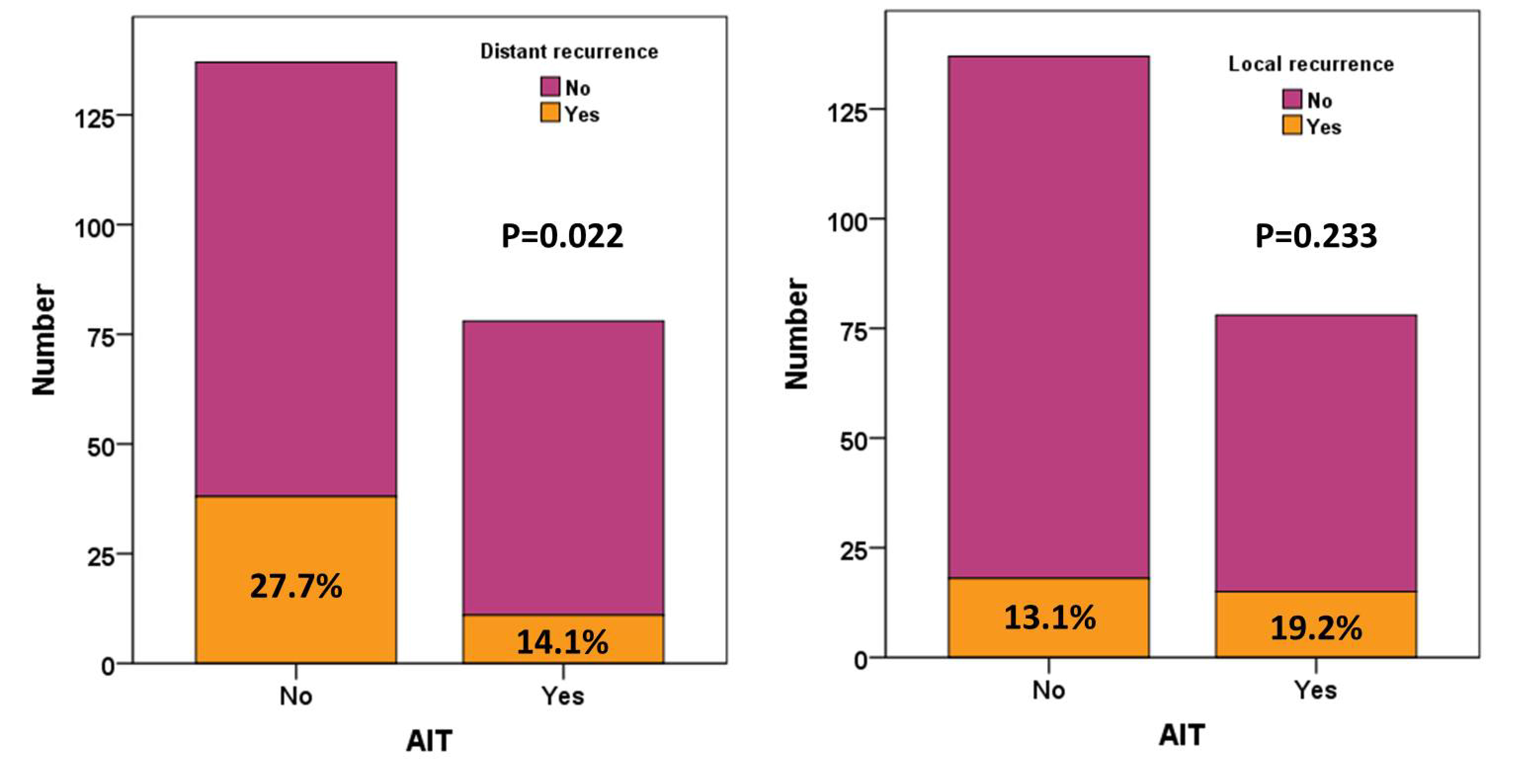

Supplement: Supplementary Figure 2 — Correlation between AIT and recurrence. AIT can effectively reduce distant recurrence (14.1% vs. 27.7%, P=0.022), but not for local recurrence (19.2% vs. 13.1%, P=0.233). [file Image2.jpeg]

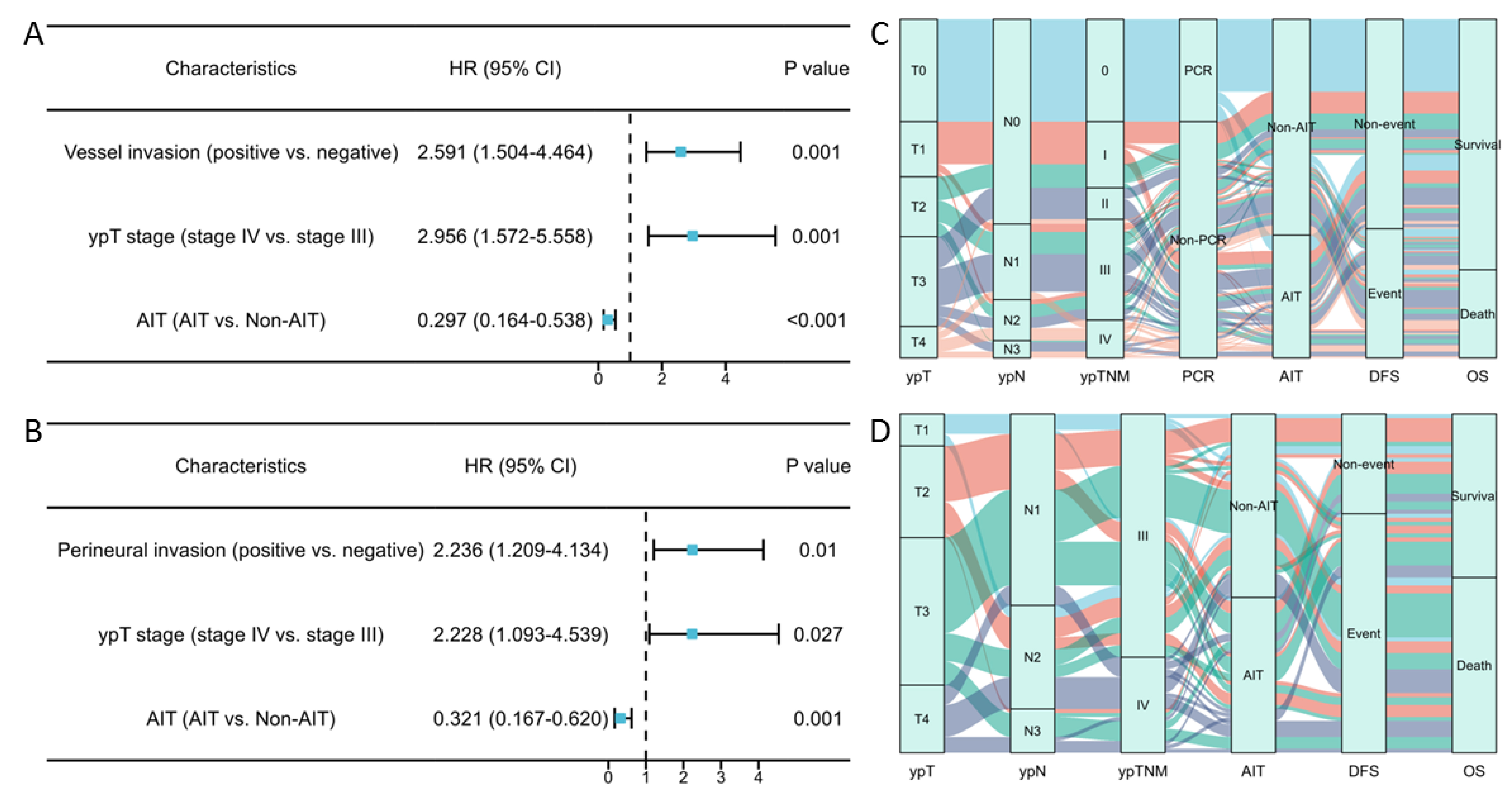

Supplement: Supplementary Figure 3 — Multivariable analysis in ypT+N+ individuals. Parameters linked to DFS (A) or OS (B) in patients with ypT+N+ ESCC. The Sankey diagrams regarding relations among AIT, ypTNM stages, and prognosis for all cohorts (C) or those with ypT+N+ (D). [file Image3.jpeg]

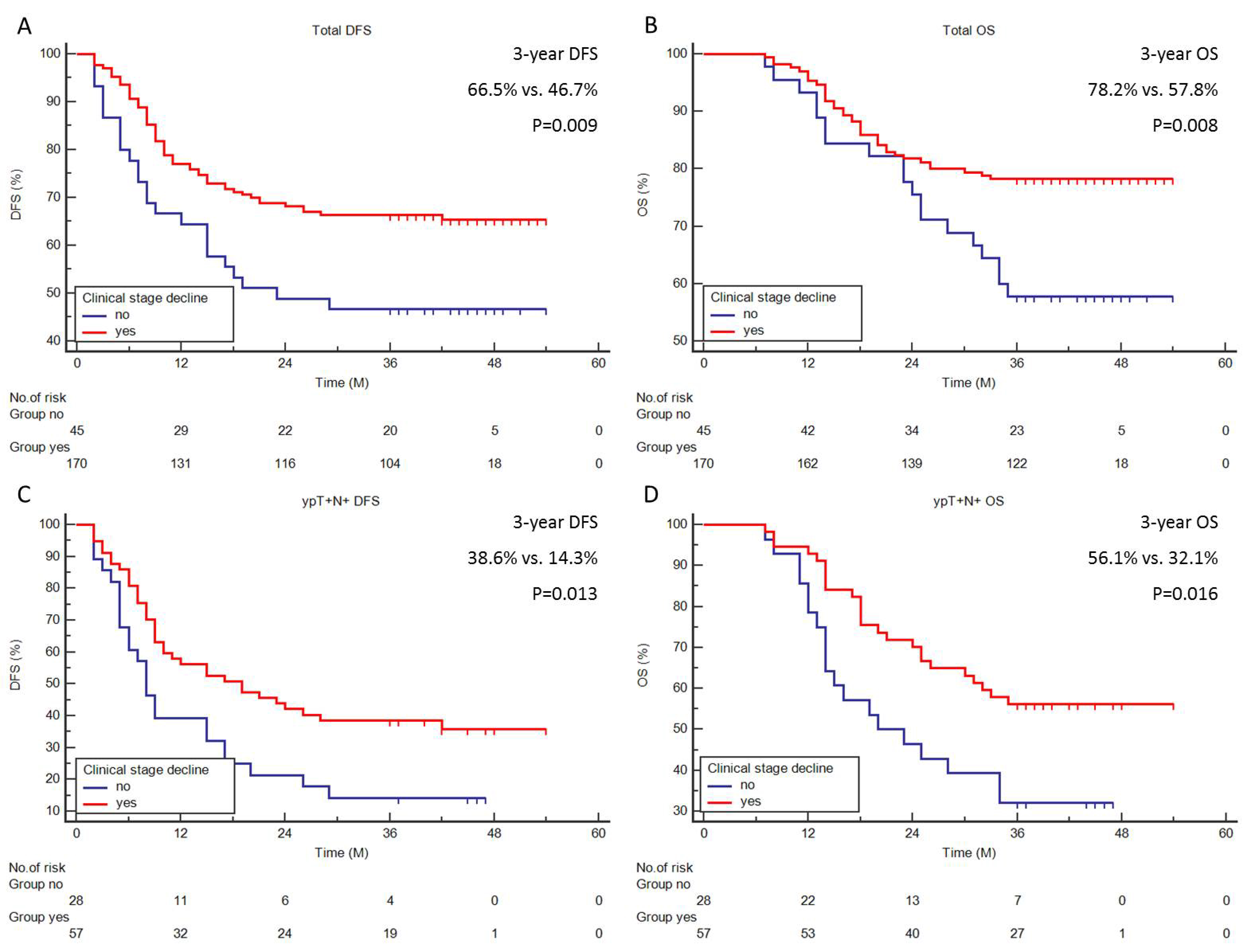

Supplement: Supplementary Figure 4 — Survival grouped by clinical stage decline. The 3-year DFS (A) or 3-year OS (B) of all cohorts with and without clinical stage decline. The 3-year DFS (C) or 3-year OS (D) in ypT+N+ patients with and without clinical stage decline. [file Image4.jpeg]
